# Assessment of the Health Status of Spanish Schoolchildren Based on Nutrimetry, Lifestyle and Intestinal Parasites

**DOI:** 10.3390/nu15122801

**Published:** 2023-06-19

**Authors:** Estephany Tapia-Veloz, Marisa Guillén, María Trelis, Tannia Valeria Carpio-Arias, Mónica Gozalbo

**Affiliations:** 1 Area of Parasitology, Department of Pharmacy and Pharmaceutical Technology and Parasitology, University of Valencia, 46010 Valencia, Spain; e.tapiaveloz@gmail.com (E.T.-V.); maria.trelis@uv.es (M.T.); 2 Department of Medicine and Public Health, Science of the Food, Toxicology and Legal Medicine, University of Valencia, 46010 Valencia, Spain; marisa.guillen@uv.es; 3 Joint Research Unit on Endocrinology, Nutrition and Clinical Dietetics, University of Valencia-Health Research Institute La Fe, 46026 Valencia, Spain; 4 Research Group on Food and Human Nutrition, Escuela Superior Politécnica de Chimborazo, Riobamba 060101, Ecuador

**Keywords:** malnutrition, nutrimetry, schoolchildren, lifestyles, intestinal parasites, risk factors

## Abstract

Malnutrition in Spanish schoolchildren, and its relationship with lifestyles, has been studied, but Nutrimetry (a nutritional status indicator), and data on intestinal parasitism and its risk factors, have never before been taken into account. A total of 206 children aged 3–11 years, from two schools in the Valencian Community, participated. Demographic characteristics, diet, lifestyles, behavioural habits and anthropometric (weight, height) and coproparasitological data were collected. Nutrimetry was used to analyse nutritional status. Statistical analyses were performed to ascertain associations between lifestyle, selected parasite species and nutritional status. Multivariate logistic regression analysis was used to assess the strength of the association of the suspected risk factors with the presence of intestinal parasitism. The prevalence of overweight was 32.6%. A total of 43.9% had a high adherence to the Mediterranean Diet, for which mean daily intake was 2428.7 kcal. Intestinal parasitism was identified in 49.5% of the children (*Giardia duodenalis*: 28.6%). The source of drinking water was found to be a risk factor for intestinal parasitism. No positive association between the variables analysed and nutritional status could be confirmed. Nutrimetry is a good indicator for a complete analysis of nutritional status. It highlights the prevalence of overweight. Intestinal parasitism was identified in almost half of the participants and is a variable that should not be underestimated.

## 1. Introduction

Child malnutrition continues to be a global health problem with multifactorial origins [1]. In Europe, there are epidemic proportions of overweight that constitute one of the main health issues; it is a predetermining factor for the future development of non-communicable diseases such as type 2 diabetes, hypertension and dyslipidaemia [2], with Spain being at the forefront in terms of childhood overweight and obesity [3]. Previous studies in Spanish children report overweight figures ranging from 21.2% to 45.2% [4,5,6,7,8,9]. The body-mass-index-for-age z-score (BMIZ) is an indicator used for the diagnosis of nutritional status; its use is easy, as well as its interpretation, and it is also the most widely used, but it is advisable to complement it with other indicators. For this reason, Selem-Solís et al., in 2017, developed the “Nutrimetry” method, which combines two accessible and easy anthropometric variables, the BMIZ and the height-for-age z-score (HAZ), whose intention is to facilitate a joint interpretation of these two indicators and generate a more complete diagnosis of nutritional status [10,11].

Traditional Mediterranean diets (characteristic of Euro-Mediterranean countries, based on traditional food and drink and often homemade) are associated with a low prevalence of chronic diseases and a longer life expectancy among populations with adherence to them [12]. Unfortunately, adherence to this type of diet is deteriorating [13], with a preference for high-calorie, ultra-processed products replacing vegetables and fruit [2,8,14,15]. The KidMed questionnaire is an easy and good tool to assess adherence to the Mediterranean diet (AMD) [16], but it does not take into account daily caloric and nutrient intake, a limitation that a food frequency questionnaire (FFQ) would provide. FFQs allow valid, accurate information to be obtained and are easy to administer without the need for a specialised interviewer [17,18,19]. Malnutrition problems, especially overnutrition, are directly related to sedentary lifestyles. The WHO recommends that children should spend at least one hour a day engaging in moderate- or vigorous-intensity physical activity (PA) and that screen time (ST) should be less than two hours a day [20]. However, many children do not meet these recommendations [21], similarly to what was found in the 2019 PASOS study in the Spanish population aged 8–16 years.

It is common for nutrition studies to be carried out with the variables described above, such as anthropometric, dietary and lifestyle values, leaving aside the possible causes of infectious and immunological origin that may be associated with these states of malnutrition, such as intestinal parasitism. These infections are an important child health problem that has historically affected mainly low-income countries, but is becoming more frequent in high-income countries, such as Spain, due to demographic, climatic and behavioural changes [22,23,24,25,26,27]. There are some intestinal parasite species associated with an altered nutritional status, such as the unicellulars *Giardia duodenalis* and *Cryptosporidium* spp. and the helminths *Ascaris lumbricoides* and *Trichuris trichiura* [28,29,30]. In the case of other parasites, such as amoebae, *Enterobius vermicularis* and *Blastocystis* sp., there is no evidence that they affect nutritional status and some of the species may even be considered commensal, but their presence will be related to inadequate hygienic sanitary measures [31,32] and, as they all share faecal–oral transmission, they can spread easily [33,34]. Studies carried out in Spain in recent years have estimated the prevalence of intestinal parasites in children ranging from 10.7% to 28.0% [27,32,35,36,37,38,39]. These results vary according to the study areas, as well as by the techniques used, where molecular techniques (PCR) increase the sensitivity of detection [40]. Specifically, in the school population of the Valencian Community, the study by Chover et al. in 2010 [27] is one of note, in which they found that 27.4% of the children had at least one species that was identified by light microscopy.

In the Valencian Community to date, there are studies of schoolchildren that have analysed nutritional status with the most frequent indicator (BIMZ), its relationship with the “typical variables” such as dietary habits and lifestyles, but not with intestinal parasitism, and the last existing study on the prevalence of intestinal parasitism in this type of population is from 2010 only, and was analysed with one faecal sample by optical microscopy and without analysing the risk factors of parasitism. In view of the above, the aim of this research is to evaluate the nutritional status of schoolchildren attending primary schools in the Valencian Community with a different indicator such as Nutrimetry, and the relationships with eating habits (KidMed and FFQ), lifestyles (PA and ST) and parasite species that may alter it, and to analyse the possible risk factors associated with the presence of intestinal parasitism.

## 2. Materials and Methods

### 2.1. Ethics Considerations

This research has the approval of the Human Research Ethics Committee of the University of Valencia (the procedure number is H1518738039128 (date: 1 March 2018)), in order to meet the principles of the Helsinki Declaration and obey Spain’s legislations on biomedical research and protection of personal data.

### 2.2. Study Design and Setting

A prospective cross-sectional study was carried out involving 206 schoolchildren, aged between three and eleven years, in the Valencian Community who attended two schools, one in La Cañada (Valencia) and another in Vila-Real (Castellón). Data and samples were collected from participating schoolchildren during 2019–2020. The municipalities to which the public schools belonged were very similar in terms of socioeconomic and socio-demographic characteristics (Table S1, [41,42]).

### 2.3. Recruitment and Sampling

One school per zone was selected that met the following criteria: (1) the number of students enrolled had to be greater than 200, (2) the school had to be of a medium-high socioeconomic level and (3) there had to be commitment and involvement in all phases on the part of the teachers and directors of the institution. School principals were contacted and invited to participate on a voluntary basis in order to achieve the third point. A total of 113 schoolchildren were recruited in Vila-Real and 96 in La Cañada.

Informative meetings were personally held with teachers, who explained the goals and procedures of the project to schoolchildren. This phase lasted three months. Meanwhile, the school principal sent a voluntary invitation letter and information to all parents in their children’s school diaries. Prior to the information and sample collection phase, informed consent forms were obtained and signed by parents for all participating children. No financial remuneration was given for being part of the research. Once all the weight and height data and samples were processed and analysed, a report was personally delivered to each parent in a sealed envelope.

### 2.4. Data Collection

Five nutritionists were trained to collect data and samples. The information collected followed previously standardized protocols. Anthropometric assessment, a standardised questionnaire and a coproparasitological assessment were carried out as indicated below:

(a) Anthropometric Assessment

At the time of weighing, the participants wore the least amount of clothing possible, and an Omrom^®^ (Hoofddorp, The Netherlands) electronic scale with an accuracy of 100 g was used. Height was measured without shoes and socks and was taken with a Seca216^®^ (Hamburgo, Germany) mechanical measuring rod with a precision of 1 mm.

**Figure 1:**
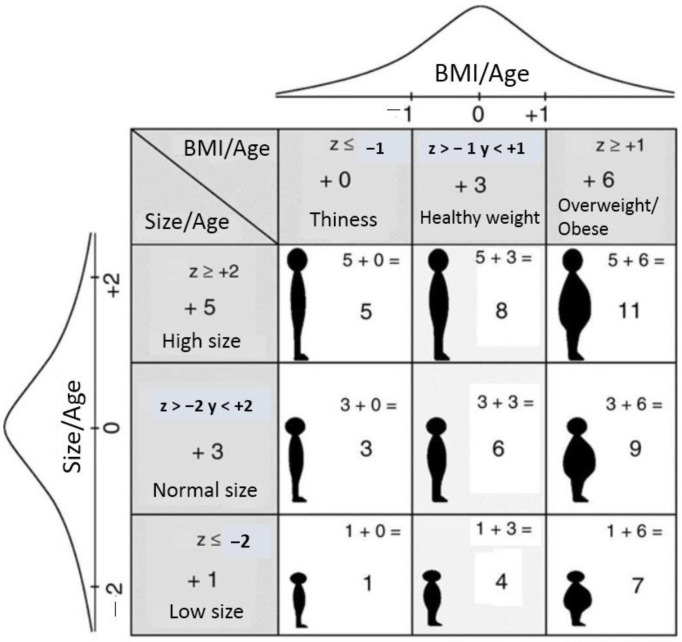
Nutrimetry information as the joint interpretation of HAZ and BMIZ (Tapia-Veloz et al., 2022) [44].

Anthropometric data, date of birth and gender were entered into the computer programs WHO Anthro for under-fives and WHO AnthroPlus for over-fives (WHO 2009; Anthro for Personal Computers, Version 3.01: Software for Assessing Growth and Development of the World’s Children). This obtained HAZ and BMIZ and classified participants into different types and degrees of malnutrition [43]. Nutrimetry combines these two indicators in a three-by-three table (Figure 1) and facilitates a final joint interpretation of nutritional status.

**Figure 2:**
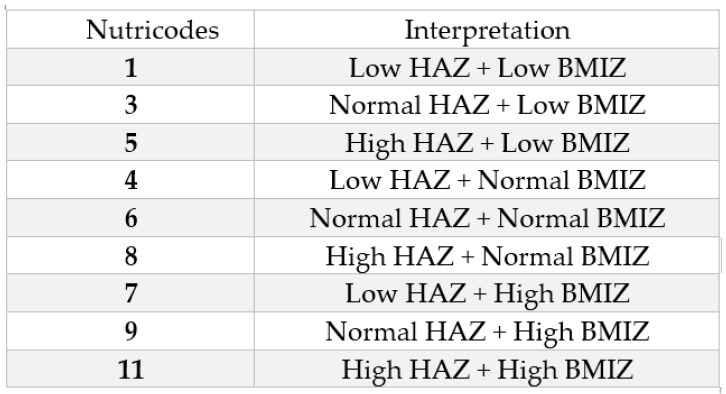
Nutricodes (Tapia-Veloz et al., 2022) [44].

The nine nutricodes generated by the combination of HAZ and BMIZ can be seen in Figure 2, and represent the diagnosis of nutritional status [10].

(b) Questionnaire

Questions included: (b.1) demographic characteristics (age, gender, locality of residence, number of relatives residing at home); (b.2) eating habits (KidMed questionnaire [16], FFQ [45]); (b.3) lifestyles (physical activity, use of screens); and (b.4) behavioural habits (source of drinking water, hand and fruit/vegetable washing, contact with domestic animals, playing outdoors). The participants returned the completed questionnaires, together with the signed informed consent forms, for collection, as described above (Table S2).

(c) Coproparasitological Assessment

To collect stool samples, the participating children whose parents signed the informed consent were given uniquely labelled kits (10 mL polystyrene plastic tube with 5 mL 70% ethanol inside, disposable spatula, Graham tape and instructions). Parents assisted the children to collect them and delivered them to the school the next day. Collected stool samples were immediately transported to the Parasitology Laboratory of the University of Valencia and stored at 4 °C until further processing. A total of 206 samples were collected and processed. One stool sample per child was collected. A 3 g stool sample from each participant was concentrated and filtered for 5 min at 2500 rpm using Midi Parasep^®^ filter devices (Apacor Ltd., Wokingham, UK). From the sediment obtained, a part was used for DNA extraction (see below) and, to the remaining part, a few drops of 10% formalin was added in a 1:3 ratio for microscopy analysis. With a small amount of the remaining sediment, thin smears were also prepared and stained with the Modified Ziehl-Neelsen (MZN) for the detection and identification of *Cryptosporidium* spp. and other coccidian protozoa. Graham tapes were examined by 10× microscopy for detecting eggs of *Enterobius vermicularis*. All stool samples were observed microscopically at 10× and 40× in order to detect structures such as eggs, cysts, oocysts, trophozoites and larvae. If none were found, it was considered negative. DNA was extracted and purified from all samples in order to perform molecular techniques to complement the microscopic observation. Genomic DNA was isolated from 200 mg of each stool sample using the QIAamp DNA Stool Mini Kit (Qiagen, Hilden, Germany) according to the manufacturer’s instructions. Extracted and purified DNA samples were eluted in 100 μL of PCR-grade water and kept at 4 °C until further molecular analysis. The presence of *G. duodenalis* was investigated using a real-time PCR (qPCR) targeting a 62-bp region of the small subunit of the rRNA (*ssu* rRNA) gene of the parasite [46]. The final mixing reaction volume was 15 μL, a commercial assay with the specific primers and probe (LightMix Modular Assays Giardia; Roche^®^, Basel, Switzerland), together with mastermix (dNTPS, buffer, thermostable Taq polymerase) (PerfeCTa qPCR ToughMix; Quanta Biosciences, Gaithersburg, MD, USA), was used. An amount of 5 μL of sample and positive control DNA was added to each well, to which the negative control water was added. The StepOnePlus thermcycler qPCR^®^ (Applied Biosystems^®^, Foster City, CA, USA) was used. Samples that amplified before cycle 43 were considered positive. *Blastocystis* sp. was identified through a direct PCR protocol, targeting a 600 bp fragment of the *ssu* rRNA gene [47,48]. In the case of *Cryptosporidium* spp., a nested PCR protocol was used to amplify a 587 bp fragment of the *ssu* rRNA gene of this parasite [49]. Detailed information on the oligonucleotides used for the molecular identification of the unicellular parasites investigated in this study is presented in Table S3.

### 2.5. Data Analysis

Descriptive statistics were calculated, including measures of central tendency (mean and median), measures of dispersion (standard deviation, range and coefficient of variation) and measures of shape (asymmetry and pointing) for quantitative variables, as well as the absolute and relative frequencies for the qualitative variables. To see a possible heterogeneity of the results, an analysis of association, stratified by age and gender, was carried out. The Mann–Whitney U test and Fisher’s exact test (non-parametric tests) were used when the data were stratified and their sample size was less than 15. To test the null hypotheses of no association between the risk of intestinal parasitism and the lifestyle habits controlled by confounders or effect modifiers, multiple logistic regression analyses, with dummy variables for categorical terms, were applied. Different models were tested. Analyses with odds intestinal parasitism as an outcome (yes/no) were always adjusted by primary school of origin. Covariates used in the models were demographic data and the traditional hygiene measures (washing hands, washing fruits and vegetables, contact with domestic animals and sources of drinking water such as bottled, tap and osmosis water). All the covariates were categorical variables. The magnitude of the association was expressed as adjusted odds ratios (AORs) with a 95% confidence interval (CI). A *p*-value ≤ 0.05 was considered significant. All the variables were analysed using SPSS software version 26.0 (Statistical Package for Social Sciences, Chicago, IL, USA).

## 3. Results

The participants were schoolchildren from two primary schools in the Valencian Community (Spain). A total of 206 schoolchildren (40.3% girls), aged three to eleven years (mean: 8.1 ± 2.1 years), participated. 

Totals of 31.6% (65/206), 1.5% (3/206) and 1.0% (2/206) of the total children corresponded to nutricodes 9, 3 and 11 (associated with malnutrition), respectively, highlighting the high prevalence of nutricode 9, which corresponds to a normal height and overweight. Nutricodes 1, 4, 5 and 7 were non-existent. The nutritional status stratified by gender and age, and assessed by Nutrimetry, of the participants is detailed in Table 1. No statistical differences were found between the two stratified variables and the distribution of nutricodes (*p* ≤ 0.05).

Regarding the AMD evaluated by the KidMed questionnaire, low AMD was found in 7.3% of all children, medium AMD in 48.8% and high AMD in 43.9%. When we stratified by gender, differences were found (*p* = 0.037) whereby boys presented higher numbers of high AMD compared to girls (70%, 63/90 vs. 30%, 28/90). The results of nutritional status by Nutrimetry and AMD are shown in Table 2; we did not find any statistical difference by nutricodes (*p* ≤ 0.05).

Regarding FFQ analysis, in the participating boys, the mean kcals consumed per day from their total diet, and kcals derived from carbohydrates (CHO), protein and fat were 2428.7 kcal (SD: 548.9 kcal), 1392.7 kcal (SD: 298.8 kcal), 113.3 kcal (SD: 30.4 kcal), and 923.1 kcal (SD: 248.7 kcal); for girls, these were 2372.1 kcal (SD: 528.1), 1365.2 kcal (SD: 281.4 kcal), 108.3 kcal (SD: 25.4 kcal) and 898.5 kcal (SD: 249.1 kcal). No differences were found according to sex (*p* ≤ 0.05). The frequency according to the Nutrimetry codes for daily intake in kcals of total diet, CHO, protein, and fat in the surveyed schoolchildren are shown in Table 3, and no statistical differences by nutricodes were obtained.

When we analyse the prevalence of other lifestyle habits in the enrolled population, 72.3% and 30.6% of the participants did not comply with recommendations for PA (at least one hour per day) and ST (less than two hours per day), respectively. We did not find any differences between gender or age and also with nutritional status.

Intestinal parasitism was also evaluated in all the participants (Table 4). Overall, 49.5% (102/206) of the participating schoolchildren were infected/colonised by at least one intestinal parasite species and 14.6% (30/206) had two or more species. The four species identified were *G. duodenalis* (28.6%; 59/206), *Blastocystis* sp. (19.4%; 40/206), *E. vermicularis* (19.4%; 2/206) and *Entamoeba hartmanni* (1.0%; 2/206). No statistical differences in intestinal parasitism were found when stratifying by gender and age groups. Of the species identified, *G. duodenalis* may affect the nutritional status of children, so we analysed this parasite and the two most prevalent nutricodes (6 and 9). We found no statistical differences (*p* = 0.369) of the participants corresponding to nutricode 6 and 9 where 27.4% and 30.8% had *G. duodenalis*, respectively. Regarding the traditional risk factors for intestinal parasitism, the prevalences were as follows: 52.9% had contact with animals; 6.3% did not wash fruits/vegetables before consumption; 34.5% and 51.0% did not wash their hands before eating and after bathing, respectively; 64.1%, 9.7% and 26.2% consumed bottled, tap and osmosis water, respectively; and, in 12.6%, there were five or more relatives residing at their home.

Finally, to assess the risk of intestinal parasitism as a function of possible risk factors (classic or traditional risk factors) in the studied population, a multivariate logistic regression analysis was carried out (Table 5).

The only variable that was significantly related to the risk of intestinal parasitism was the source of drinking water. The schoolchildren who consume tap water had significantly greater odds of having intestinal parasitism compared to those who consume bottled water (OR = 3.263; CI 95%: 1.076–9.987; *p* = 0.037). Surprisingly, those who consume water filtered by osmosis did not show significantly lower odds (OR = 0.522; CI 95%: 0.264–1.032; *p* = 0.061). When other multivariate logistic regression models using demographic and/or lifestyle variables (adjusted and non-adjusted) were tested no significant associations were found. 

## 4. Discussion

We studied the nutritional status of schoolchildren using Nutrimetry, the first time this indicator has been used in the Spanish population. Previous research only used the BMIZ as an indicator [7,50,51,52,53,54]. The prevalence of overweight in our research (codes 7, 11) was 32.6% and the prevalence of thinness (code 3) was 1.5%. Previous studies in similar populations reported figures in line with those found, with overweight ranging from 21.2% to 45.2% [4,5,6,7,8,9] and underweight from 0.4% to 13.3% [7,54]. According to the National Health Survey (2017) of the Spanish Ministry of Health, in the Valencian Community, the prevalence of overweight was 28.2% [55], a number that, compared to the one we found (32.6%), confirms its increase. These data are also in accordance with the WHO, which states that, among children and adolescents (5 to 19 years of age), overweight and obesity have increased dramatically in recent years [56]. Excess weight in schoolchildren is clearly an urgent public health problem to be tackled.

Although the majority of participants had a nutritional status that was in codes 6 and 9, and while the remaining codes (1, 3, 4, 5, 7, 8 and 11) had very few or no children, this is a novel indicator because it would facilitate comparisons with other populations. Nutrimetry allows for a more detailed and in-depth description and analysis of the population distribution of malnutrition by combining two indicators, that are normally used separately, to obtain a single diagnosis.

It does not require specialised software to generate the nine subgroups. It uses the WHO growth curves as a reference and can therefore be considered a suitable anthropometric indicator. Furthermore, it uses neutral language (numbers) to report the results, which facilitates communication without an emotional semantic load by avoiding the use of words such as “fat” or “skinny” [10,11,43].

We found that 7.3%, 48.8% and 43.9% of the children had low, medium and high AMD, respectively. These percentages obtained are in agreement with other studies in similar populations [9,15,57]. There are studies that found statistical differences between AMD and nutritional status, where having high adherence is a protective factor against excess weight [58,59,60,61], but there are also investigations that did not find statistical differences [9,15,57,62,63]. In the case of our work, we found no differences. It is important to note that KidMed does not take into account calories consumed and, therefore, a high adherence does not guarantee a healthy nutritional status in its entirety, which is why we included the FFQ.

The mean age of the schoolchildren was 8.1 years (±2.1 years); EFSA recommends 1815 kcal for boys and 1696 kcal for girls per day at this age, with CHO being between 45 and 60% of the total, protein being between 0.75 and 0.92 g/kg and fat being between 20 and 35% of the total [64]. The results obtained from the FFQ analysis are higher than the recommended values, revealing a possible overestimation. The long FFQ, the one we used, has the disadvantage of overestimating the total kcals and nutrients consumed [64,65,66,67]. This is due to the greater effort and time required to answer the questionnaire, the sum of consumption frequencies due to the existence of many items, the errors in the estimation of the frequency and the perception of the size of portions may be greater than the real ones [68,69,70,71,72], especially considering that the respondents were the legal representatives. There was no association between nutritional status and mean total kcals and macronutrient concentrations; we also believe this is due to the overestimation of the FFQ. Healthy eating is an essential factor, but must be accompanied by lifestyles that include daily PA in children (of at least one hour of moderate/vigorous intensity) and less than two hours in front of screens in order to ensure proper health [20,73]. PA at this age is favourably associated with indicators such as adiposity, bone and skeletal health, cardiometabolic health and cognitive and motor development [20]. In the PASOS study (2019) carried out on 38,887 Spaniards aged 8 to 16 years, 63.3% and 45.6% did not comply with the recommendations for PA and ST, respectively [74]; these are similar data to those that we found (72.3% and 30.6%, respectively).

Although no association was found between nutritional status and the two methods used to assess dietary intake (FFQ and AMD), the prevalence of overweight is high and most participants did not meet the daily PA recommendations. For overweight/obesity to exist, there must be an incorrect imbalance between the energy that is spent and consumed for their age. Response bias could be an important factor that may have led to not finding an association, but this does not rule out it being a reality. In order to avoid this limitation for future studies, an explanation of portion size could be strengthened, an additional method, such as a 24 h reminder, could be included and a second FFQ could be used to allow a comparison. 

The high prevalence of intestinal parasitism has been associated with tropical/subtropical countries in association with poverty [75]. This reality is changing in areas with different economic and climatic characteristics, such as Spain, where numbers are increasing. The reasons for this include migration from endemic countries, increased international travel, consumption of fresh food and fostering/adoption of children from these areas, more consumption of organically grown food and the climate crisis [22,23,24,25,26,27]. In the present study, 49.5% of the participants had intestinal parasitism, a high number compared to other studies which reported between 10.7% and 28.0% [27,32,35,36,37,38,39]. Chover et al. (2010) [27], in their work on schoolchildren in Valencia, identified *Blastocystis* sp. (14.9%), *E. vermicularis* (9.6%) and *G. duodenalis* (6.1%) as the most prevalent species; in our case, these were found in 28.6%, 19.4% and 19.4% of participants, respectively. Our numbers are probably higher due to an increase in intestinal parasitism, but also due to the combination of light microscopy and molecular techniques that stand out as being more sensitive [40]. When analysing the relationship between Nutrimetry and *G. duodenalis*, we did not find a significant association, this is possibly because the impact towards nutritional status usually occurs when there is undernutrition and, in our research, this condition was very low. Even so, its presence should not be underestimated. The number of cases of *G. duodenalis* was high and the similarity of its prevalence with a study in children conducted in an endemic area such as Ecuador (30.8%) is striking. It is possible that some of these participants were asymptomatic, which facilitates the spread of the parasite when not treated [33,76]. This species can affect the intestinal barrier and can be a triggering factor for secondary food intolerance/malabsorption, dyspepsia or irritable bowel syndrome [30,34,77].

The factors we took into account as risk factors associated with intestinal parasitism in children are in line with those studied by other researchers, who found no association between these variables and being parasitized [32,36,78]. Our multivariate logistic regression analysis revealed that the only variable associated with intestinal parasitism was the source of drinking water. Schoolchildren who consumed tap water were significantly more likely to be parasitized compared to those who consumed bottled water. Those who consumed water filtered by osmosis did not show significantly lower odds. These data should be taken with caution, especially considering that the number of participants who consumed tap water was very low (9.7%), that some variable that we did not take into account could alter the results and the limitation of the low sample size.

Certain limitations may have compromised the accuracy of the results and conclusions of this study. The epidemiological data generated here might not be representative of the whole Valencian Community scenario, for example: the data collected in the questionnaire could lead to a certain recall/recording bias; the two questionnaires used to collect information on dietary habits should be complemented by some additional method; and, finally, some risk factors for intestinal parasitism not included could provide further analysis. Future studies should be carried out in larger samples so that they can be representative, where the analysis of intestinal parasitism should be an important variable to be taken into account together with its symptomatology. All this would provide a clearer picture in order to take measures to improve the nutrition, health, diet and lifestyle status of the school population of the Valencian Community. 

## 5. Conclusions

Nutrimetry can be an interesting indicator, that is easy to use and interpret and is suitable for use at a clinical and epidemiological level, for a more complete analysis of the nutritional status of children because it allows the obtaining of a joint diagnosis of HAZ and BMIZ that are typically used separately.

This study shows the high prevalence of overweight in schoolchildren, which confirms the alarming figures worldwide and that urgent measures need to be taken to correct it at the family, school and governmental levels. 

Lifestyles are a fundamental aspect to take into account because of their relationship with nutritional status, but it is necessary, above all at the level of dietary habits, to combine various methods that improve their analysis and facilitate the finding of associations with nutritional status. 

Regarding intestinal parasitism, the high numbers found are striking, especially when compared with previous studies in similar populations. *G. duodenalis* was the most prevalent, which can cause digestive problems in addition to malnutrition, such as the malabsorption of certain nutrients. Therefore, intestinal parasites in Spain need more attention in order to understand the associated factors and their impact on children’s health.

## Figures and Tables

**Table 1:** Frequencies (%) of the participating children according to the nutricodes stratified by gender and age.

	BOYS (*n* = 123)			* *p*-Value	GIRLS (*n* = 83)			* *p*-Value	** *p*-Value
	<5	5–8	≥9		<5	5–8	≥9		
Nutricode	(*n* = 5)	(*n* = 67)	(*n* = 51)		(*n* = 6)	(*n* = 35)	(*n* = 42)		
Thinness				0.918				0.807	0.883
1	0 (0)	0 (0)	0 (0)		0 (0)	0 (0)	0 (0)		
3	0 (0)	1.5 (1)	0 (0)		0 (0)	2.9 (1)	2.4 (1)		
5	0 (0)	0 (0)	0 (0)		0 (0)	0 (0)	0 (0)		
Normal Weight									
4	0 (0)	0 (0)	0 (0)		0 (0)	0 (0)	0 (0)		
6	60 (3)	64.2 (43)	66.7 (34)		66.7 (4)	62.8 (22)	69.0 (29)		
8	0 (0)	0 (0)	0 (0)		0 (0)	2.9 (1)	0 (0)		
Overweight and Obesity									
7	0 (0)	0 (0)	0 (0)		0 (0)	0 (0)	0 (0)		
9	40 (2)	34.3 (23)	33.3 (17)		33.3 (2)	25.7 (9)	28.6 (12)		
11	0 (0)	0 (0)	0 (0)		0 (0)	5.7 (2)	0 (0)		

The number of children is shown in brackets. * *p*-value for the comparison of the distribution of the frequencies of the codes established by Nutrimetry and age; ** *p*-value for the comparison of the distribution of the frequencies of the codes established by Nutrimetry and gender.

**Table 2:** Frequencies (%) of the participating children according to the nutricodes stratified by the AMD (KidMed questionnaire).

Nutricode	Low AMD (*n* = 15)	Medium AMD (*n* = 100)	High AMD (*n* = 90)	* *p*-Value
Thinness				
1	0 (0)	0 (0)	0 (0)	
3	0 (0)	3.0 (3)	0 (0)	
5	0 (0)	0 (0)	0 (0)	
Normal weight				
4	0 (0)	0 (0)	0 (0)	0.611
6	66.7 (10)	69 (69)	62.2 (56)	
8	0 (0)	0 (0)	1.1 (1)	
Overweight and obesity				
7	0 (0)	0 (0)	0 (0)	
9	33.3 (5)	27 (27)	36.7 (33)	
11	0 (0)	1.0 (1)	1.1 (1)	

Number of children is shown in brackets. * *p*-value for the comparison of the distribution of the frequencies of the codes established by Nutrimetry and the AMD.

**Table 3:** Daily intake of total kcals, and CHO, protein and fat (kcals) of the participating children stratified by the nutricodes.

Nutricode	% (*n*)	TOTAL kcal			CHO (kcal)			PROTEIN (kcal)			FAT (kcal)		
		Mean	Sd	* *p*-Value	Mean	Sd	* *p*-Value	Mean	Sd	* *p*-Value	Mean	Sd	* *p*-Value
Thinness													
1	0 (0)			0.45			0.37			0.21			0.69
3	1.5 (3)	2391.9	459.3		1354.4	264.6		108.1	21.5		929.4	228.1	
5	0 (0)												
Normal Weight													
4	0 (0)												
6	65.5 (135)	2357.5	480.7		1353.8	272.4		108.3	26.1		895.9	212.6	
8	0.5 (1)	2849.8	-		1640.0	-		142.1			1067.8		
Overweight and Obesity													
7	0 (0)												
9	31.6 (65)	2498.8	651.9		1435.1	329.9		117.0	33.1		946.7	313.7	
11	0.9 (2)	2458.1	341.5		1436.6	82.9		125.9	9.4		895.5	249.2	

Number of children is shown in brackets. * *p*-value for the comparison of the distribution of the frequencies of the codes established by Nutrimetry and the total kcal intake, and kcal intake derived from CHO, protein and fat. CHO: carbohydrates.

**Table 4:** Frequencies (%) of the studied population according to the presence of total intestinal parasitism, multiparasitism and detected species analysed by optical microscopy and PCR.

	TOTAL
Detected Species	(*n* = 206)
*G. duodenalis*	28.6 (59)
*Blastocystis* sp.	19.4 (40)
*E. vermicularis*	19.4 (40)
*E. hartmanni*	1.0 (2)
Total intestinal parasitism	49.5 (102)
Total intestinal multiparasitism	14.6 (30)

Number of children is shown in brackets.

**Table 5:** Multivariate logistic regression analysis to estimate the risk of intestinal parasitism in the participating children.

Variable	Category	*n*	Intestinal Parasitism		
			AOR	95% CI	* *p*-Value
Gender	Girl	83	1.352	0.738–2.479	0.329
	Boy	123	Ref.	-	-
Age group	3 to <5 years	11	2.495	0.572–10.882	0.224
	5 to <9 years	102	0.719	0.382–1.354	0.307
	9 to <12 years	93	Ref.	-	-
Contact with animals	Yes	97	1.111	0.609–2.028	0.731
	No	109	Ref.	-	-
Washing fruits/vegetables	Yes	193	Ref.	-	-
	No	13	0.853	0.246–2.963	0.802
Handwashing before eating	Yes	135	Ref.	-	-
	No	71	1.293	0.659–2.536	0.455
Handwashing after bathing	Yes	101	Ref.	-	-
	No	105	0.823	0.435–1.557	0.549
Source of drinking water	Bottled	132	Ref.	-	-
	Tap	20	3.263	1.076–9.897	0.037
	Osmosis	54	0.522	0.264–1.0320	0.061
Plays outdoors	Yes	147	0.965	0.481–1.939	0.921
	No	59	Ref.	-	-
Number of relatives residing at home	2–4 people	180	Ref.	-	-
	≥5	26	0.430	0.167–1.109	0.081

AOR: adjusted odds ratio. CI: confidential interval. * Statistical significance was set at α = 0.05. Ref: category of reference.

## Data Availability

The data sets generated or analyzed during the current study are available from the corresponding author upon reasonable request.

## Supplementary Materials

The following supporting information can be downloaded at: https://www.mdpi.com/article/10.3390/nu15122801/s1, Table S1: Socioeconomic and socio-demographic characteristics of the municipalities of the two participating public schools; Table S2: English version of the standardised epidemiological questionnaire used in this study; Table S3: Oligonucleotides used for the molecular identification and/or characterization of the intestinal protists parasites investigated in the present study.
