# Supplementary material for: Assessment of the Health Status of Spanish Schoolchildren Based on Nutrimetry, Lifestyle and Intestinal Parasites
Source: Nutrients. 2023 Jun 19;15(12):2801. doi: 10.3390/nu15122801 (PMC10304592; doi:10.3390/nu15122801)
Supplement: Supplementary file 1 [file nutrients-15-02801-s001.zip › nutrients-2408342-supplementary.pdf]

Table S1. Socioeconomic and socio-demographic characteristics of the municipalities of the two participating public schools.

Variable	Municipality		Reference
	Paterna (La Cañada)	Vila-Real	
**Country**	Spain	Spain	[41,42]
**Autonomous community**	Valencian Community	Valencian Community	
**Province**	Valencia	Castellón	
**Population in relation to the community (%)**	1.41	1.01	
**Children under 16 years (%)**	19.27	16.69	
**Older than 64 years (%)**	14.68	17.05	
**Illiterate and not educated (%)**	8.24	10.79	
**Foreigners (number of people)**	7314	6791	
**Activity rate (%)**	71.72	65.66	
**Average budget per inhabitant (euros/inhabitant)**	891.69	1052.07	
**Telephone lines (no. telephone lines x 100 inhabitants)**	45.30	37.44	
**Passenger cars (no. of passenger cars x 100 inhabitants)**	51.80	52.51	
**Average cadastral value (euros)**	81,585.32	76,623.49	
**Total number of enterprises**	5969	3277	
**Surface area of the municipality (hectares)**	3585	5512	
**Urban Area (hectares)**	1211.39	1183.23	

Table S2. English version of the standardised epidemiological questionnaire used in this study.

**Variable**	**Category and State Code**
School sample	String variable (free text)
Sampling kit ID	String variable (free text)
Sampling date	Date variable
Country of birth	Sampling date
Place of residence	Sampling date
Weight	Decimal variable (number in kilograms)
Height	Decimal variable (number in centimetres)
Sex	Girl (1)<br>Boy (2)
Age	Integer variable (number)
Number of relatives residing at home	Integer variable (number)
Source of drinking water	Bottled (1)<br>Tap (2)<br>Osmosis (3)
Hand and fruit/vegetable washing	Yes (1)<br>No (2)
Contact with domestic animals	Yes (1)<br>No (2)
Playing outdoors	Yes (1)<br>No (2)
Get at least one hour of physical activity every day	Yes (1)<br>No (2)
Screen time is less than two hours per day	Yes (1)<br>No (2)

KidMed Questionnaire [16]:

**Adherence to the MEDITERRANEAN DIET in childhood**	**yes/no**
Have a piece of fruit or natural juice every day.	
Have a 2nd piece of fruit every day.	
Eat fresh (salads) or cooked vegetables regularly once +1 time a day.	
Eat fresh or cooked vegetables regularly more than +1 time per day.	
Eat fish regularly (at least 2-3 times a +1 week).	
Goes once or more per week to a fast food centre (hamburger joint).	
Likes pulses and eats them more than once a week.	
Eats pasta or rice almost every day (5 days or more per week).	
Eats a cereal or derivative (bread, etc.) for breakfast.	
Eats nuts and dried fruit regularly (at least 2-3 times a week).	
Olive oil is used at home.	
No breakfast.	

Have a dairy product for breakfast (yoghurt, milk, etc.).	
Eat industrial pastries, biscuits or cakes for breakfast.	
Eat 2 yoghurts and/or 40 g cheese every day.	
Eat sweets and/or candies several times a day.	

FFQ (the original Spanish version is provided) [45]:

Indicate with an X which corresponds to the consumption of the foods listed for the child:

	7	6	5	4	3	2	1	0
**I. LÁCTEOS**	**Más 1/día**	**1 vez/día**	**5-6/ semana**	**3-4/ semana**	**1-2/ semana**	**< 1/semana**	**< 1 vez/mes**	**Nunca**
Leche entera (1 vaso/taza)								
Leche semi o descremada (1 vaso)								
Leche condensada (1 cuharada)								
Café con leche (1 vaso)								
Café cortado con leche								
Yogur entero (1=125g)								
Yogur semi o desnatado (1=125g)								
Requesón/cuajada/queso fresco (100g)								
Queso cremoso/porciones (50g)								
Queso curado tipo manchego (50g)								
Flan (uno)								
Natillas o similares (uno)								
Horchata (1 vaso)								
Batido de soja (1 vaso)								

	7	6	5	4	3	2	1	0
**II. DULCES Y BOLLERÍA**	**Más 1/día**	**1 vez/día**	**5-6/ semana**	**3-4/ semana**	**1-2/ semana**	**< 1/semana**	**< 1 vez/mes**	**Nunca**
Galletas tipo María (1 galleta)								
Galletas de chocolate (1 galleta)								
Ensaimada/croissant (uno)								
Donuts (uno)								
Magdalenas/bizcochos (uno)								
Tartas/pastelillos (1 ración)								
Chocolate con leche (1 trozo)								
Chocolate puro (1 trozo)								
Chocolate en polvo (una cucharada desayuno)								

	7	6	5	4	3	2	1	0
**III. PAN Y CEREALES**	**Más 1/día**	**1 vez/día**	**5-6/ semana**	**3-4/ semana**	**1-2/ semana**	**< 1/semana**	**< 1 vez/mes**	**Nunca**
Pan blanco (media barra. 125g)								
Pan integral (media barra 125g)								
Pan de molde (1 rebanada)								
Rosquilletas (3-4 unidades)								
Macarrones/espagueti (1 plato)								
Paella (1 plato)								
Arroz, no paella (1 plato)								
Sopa fideos/letras (1 plato)								

	7	6	5	4	3	2	1	0
**IV. HUEVOS, CARNES, PESCADOS**	**Más 1/día**	**1 vez/día**	**5-6/ semana**	**3-4/ semana**	**1-2/ semana**	**< 1/semana**	**< 1 vez/mes**	**Nunca**
Un huevo hervido								
Un huevo frito								
Un huevo en tortilla								
Una ración pollo plancha								
Una ración pollo frito								
Un filete ternera plancha								
Un filete ternera frito o guisado								
Carne de cordero								
Una ración carne cerdo (lomo, solomillo…)								
Una ración carne de conejo (130 g)								
Una ración hígado de cordero, cerdo, ternera								
Una ración de vísceras (callos, sesos…)								
Una hamburguesa de pollo/pavo								
Una hamburguesa ternera								
Una loncha de jamón serrano (25 g)								
Una loncha de jamón de york (25 g)								
Una ración de salchichón o chorizo								
Una salchicha/longaniza fresca								
Una morcilla								
Una ración de tocino,/bacon								
Foie-gras o patés (una ración)								
Calamares/sepia a la romana (1 ración)								
Un plato de pescado frito								
Un plato de pescado plancha o hervido								
Una lata de atún en conserva								
Una lata de sardinas en conserva								
Pescados en salazón o conserva								
Una ración de almejas o mejillones								
Una ración de marisco (gambas, cigalas...)								

	7	6	5	4	3	2	1	0
**V. VERDURAS Y PATATAS**	**Más 1/día**	**1 vez/día**	**5-6/ semana**	**3-4/ semana**	**1-2/ semana**	**< 1/semana**	**< 1 vez/mes**	**Nunca**
Lechuga/escarola en ensalada (125g)								
Un tomate solo o ensalada								
Zanahorias en ensalada								
Un plato de zanahorias guisadas								
Una cebolla en ensalada o asada								
Una patata hervida o asada								
Una ración de patatas fritas								
Una berenjena								
Un calabacín								
Un pimiento								
Plato de alcachofas/espinacas/acelgas								
Un plato de judias verdes								
Una ración de esparrágos								
Una ración de setas o champiñones								
Un plato de guisantes cocinados								
Col, coliflor o brécoles								
Un pepino								

	7	6	5	4	3	2	1	0
**VI. LEGUMBRES**	**Más 1/día**	**1 vez/día**	**5-6/ semana**	**3-4/ semana**	**1-2/ semana**	**< 1/semana**	**< 1 vez/mes**	**Nunca**
Un plato de lentejas								
Un plato de garbanzos								
Un plato de judias blancas								

	7	6	5	4	3	2	1	0
**VII. FRUTAS Y FRUTOS SECOS**	**Más 1/día**	**1 vez/día**	**5-6/ semana**	**3-4/ semana**	**1-2/ semana**	**< 1/semana**	**< 1 vez/mes**	**Nunca**
Una naranja o mandarina								
Un plátano								
Una manzana								
Una pera								
Un melocotón o similares								
Una ración de fresas								
Una ración de cerezas								
Un Kiwi								
Sandia o melón (1 tajada)								
Una ración de piña natural								
Melocotón o piña en almibar								
Una ración de aceitunas								
Una ración de almendras o avellanas								
Una ración de cacahuetes								
Una ración de nueces								

VIII. ACEITES Y GRASAS	7<br>Más 1/día	6<br>1 vez/día	5<br>5-6/ semana	4<br>3-4/ semana	3<br>1-2/ semana	2<br>< 1/semana	1<br>< 1 vez/mes	0<br>Nunca
Aceite de oliva virgen extra (1 cucharada)								
Aceite de oliva refinado (1 cucharada)								
Aceites de girasol (1 cucharada)								
Aceite maíz (1 cucharada)								
Otros aceites vegetales (1cucharada)								
Mantequilla (1 cucharada o untada)								
Margarina (1 cucharada o untada)								
Manteca de cerdo (1 cucharada/untada)								

IX. BEBIDAS Y REFRESCOS	7<br>Más 1/día	6<br>1 vez/día	5<br>5-6/ semana	4<br>3-4/ semana	3<br>1-2/ semana	2<br>< 1/semana	1<br>< 1 vez/mes	0<br>Nunca
Un vaso de zumo de naranja natural								
Un vaso de zumo de frutas envasado								
Un vaso de coca-cola								
Un vaso de gaseosa/fanta								
Un vaso de tónica o bitter								
Un vaso de vino blanco								
Un vaso de vino tinto o rosado								
Cerveza (1 caña)								
Café solo sin azucar (1 taza)								
Café con azucar/edulcorante (1 taza)								
Té u otras infusiones sin azucar								
Té u otras infusiones con azucar								

X. OTROS ALIMENTOS	7<br>Más 1/día	6<br>1 vez/día	5<br>5-6/ semana	4<br>3-4/ semana	3<br>1-2/ semana	2<br>< 1/semana	1<br>< 1 vez/mes	0<br>Nunca
Sopas y cremas de sobre (1 plato)								
Palitos o delicias de pescado (1)								
Croquetas (1)								
Palomitas de maíz (1 paquete)								
Una bolsa de pipas de girasol								
Mayonesa (1 cucharada)								
Salsa de tomate (1 cucharada)								
Pizza								
Sal (una pizca a la comida)								
Azucar añadido (1 cucharada)								
Mermelada/miel (1 cucharada)								

Table S3. Oligonucleotides used for the molecular identification and/or characterization of the intestinal protists parasites investigated in the present study.

**Target organism**	**Locus**	**Oligonucleotide**	**Sequence (5′–3′)**	**Reference**
Giardia duodenalis	*ssu* rRNA	Probe	FAM–CCCGCGGCGGTCCCTGCTAG–BHQ1	[46]
		Gd-80F	GACGGCTCAGGACAACGGTT	[46]
		Gd-127R	TTGCCAGCGGTGTCCG	[46]
***Blastocystis* sp.**	*ssu* rRNA	BhRDr	GAGCTTTTTAACTGCAACAACG	[48]
		RD5	ATCTGGTTGATCCTGCCAGT	[48]
***Cryptosporidium* spp.**	*ssu* rRNA	CR-P1	CAGGGAGGTAGTGACAAGAA	[48]
		CR-P2	TCAGCCTTGCGACCATACTC	[49]
		CR-P3	ATTGGAGGGCAAGTCTGGTG	[49]
		CPB-DIAGR	TAAGGTGCTGAAGGAGTAAGG	[49]

ssu rRNA: Small subunit ribosomal RNA.
